# Prediction and expression analysis of G protein-coupled receptors in the laboratory stick insect, Carausius morosus

**DOI:** 10.3906/biy-1809-27

**Published:** 2019-02-07

**Authors:** Burçin DUAN ŞAHBAZ, Necla BİRGÜL İYİSON

**Affiliations:** 1 Center for Life Sciences and Technologies, Boğaziçi University , İstanbul , Turkey; 2 Department of Molecular Biology and Genetics, Faculty of Arts and Sciences, Boğaziçi University , İstanbul , Turkey

**Keywords:** GPCR, stick insect, RNAseq

## Abstract

G protein-coupled receptors (GPCRs) are 7-transmembrane proteins that transduce various extracellular signals into intracellular pathways. They are the major target of neuropeptides, which regulate the development, feeding behavior, mating behavior, circadian rhythm, and many other physiological functions of insects. In the present study, we performed RNA sequencing and de novo transcriptome assembly to uncover the GPCRs expressed in the stick insect Carausius morosus. The transcript assemblies were predicted for the presence of 7-transmembrane GPCR domains. As a result, 430 putative GPCR transcripts were obtained and 43 of these revealed full-length sequences with highly significant similarity to known GPCR sequences in the databases. Thirteen different GPCRs were chosen for tissue expression analysis. Some of these receptors, such as calcitonin, inotocin, and tyramine receptors, showed specific expression in some of the tissues. Additionally, GPCR prediction yielded a novel uncharacterized GPCR sequence, which was specifically expressed in the central nervous system and ganglia. Previously, the only information about the anatomy of the stick insect was on its gastrointestinal system. This study provides complete anatomical information about the adult insect.

## 1. Introduction

G protein-coupled receptors (GPCRs) constitute the major targets of small regulatory peptides or peptide hormones. They transduce these extracellular stimuli to intracellular molecular responses and become the key regulators of endocrine signaling. They contain conserved 7-transmembrane (7TM) helices, a variable extracellular N-terminus, and an intracellular C-terminus. They interact with the trimeric G-proteins from the intracellular site. GPCRs are the largest group of proteins in eukaryotes and are clustered in separate classes, namely rhodopsinlike Class A, secretin-like Class B1, adhesion GPCRs Class B2, glutamate receptors Class C, Frizzled receptors Class F, and Taste-2 receptors. Within these receptors, peptides interact with the GPCRs of Class A and Class B1. Our previous work identified a C type of Allatostatin receptor (AlstR) from Class A in the laboratory stick insect, Carausius morosus (Duan Sahbaz et al., 2017) . All types of AlstRs (A, B, and C) have a common function, which is inhibition of juvenile hormone (JH) secretion. However, their expression profiles differ in a stage-specific, tissuespecific, and species-specific manner. In addition, their inhibitory effect varies between species. The laboratory stick insect is an organism defined as an agricultural pest in some countries but fed as a pet in others. Its locomotion behavior is widely studied and modeled (Bläsing and Cruse, 2004; Gruhn et al., 2016; Dallmann et al., 2017) . Additionally, other peptidergic mechanisms, such as ecdysis behavior, circadian rhythm, and heartbeat frequency, are as well studied (Wadsworth et al., 2014; Marco et al., 2018) . Although the most researched mechanisms of the stick insect rely on peptidergic pathways, there is limited information on its neuropeptides and GPCRs. Even though its neuropeptidome was revealed recently (Liessem et al., 2018) , we need to uncover the GPCRs they target and activate in order to understand the mechanism of action of the neuropeptides. There are well-fitted approaches and tools for GPCR prediction from RNA sequencing (RNAseq) data. For instance, the RNAseq approach has been recently used to predict the GPCR profiles of different arthropods (Buckley et al., 2016; Guerrero et al., 2016) . These studies utilized the open reading frame and 7TM domain prediction tools to predict GPCR candidates. Then they filtered the reliable GPCRs with the help of the GPCRPred tool, which annotates and predicts GPCRs from other proteins with an accuracy of 99.5% (Bhasin and Raghava, 2004) . Therefore, we utilized the transcriptome data for identification of the GPCRome of C. morosus including all AlstRs and predicted the functions of some of these GPCRs in comparison to their expression profiles and neuropeptide ligands.

## 2. Materials and methods

### 2.1. Animals

The stick insects (Carausius morosus ) were obtained from the University of Cologne, Germany. They were kept in cages at room temperature (RT) and fed ad libitum in a 12-h light/dark cycle. Sampling was performed in daylight when the animals were least active. The adult females, which were ready to lay eggs, were sacrificed via CO 2 and cooled down in PBS at –20 °C. Dissection was performed in the presence of cold PBS. The organs were immediately put into TRIzol reagent and stored at –80 °C. For RNA sequencing, the animals were flash-frozen in liquid nitrogen, disrupted by a mortar and pestle, and stored at –80 °C.

### 2.2. RNA isolation

Frozen total animal tissues were mixed with TRIzol and the mixture was homogenized in a MagNA Lyser for 1 min. The homogenates were put in clean tubes and incubated at RT for 5 min. Centrifugation was performed at 12,000 × g for 10 min at 4 °C. The clear lysate was transferred into a clean microcentrifuge tube and chloroform was added in 1:5 ratio. The tube was mixed vigorously for 20 s and incubated at RT for 2–3 min. Centrifugation was performed at 10,000 × g for 18 min. The aqueous part was transferred into a clean microcentrifuge tube and 1 volume of 100% EtOH was added. The tube was inverted 6 times. Next, 700 µL of this sample was loaded into a NucleoSpin RNA column. Centrifugation was performed at 11,000 × g for 30 s and the flowthrough was discarded. The rest of the procedure was performed as recommended in the protocol of NucleoSpin RNA (740955.50, MN, Germany). The RNA samples were evaluated in MOPS gel electrophoresis in denaturing conditions. For RNA sequencing one animal sample was prepared and dried in RNAstable (Biomatrica) for shipping. For expression analyses, at least three adult animals were utilized in one replica, according to the mass of the organ.

### 2.3. Sequencing and de novo RNA assembly

Paired-end sequencing (2 × 100 bp) was generated by GENEWIZ Inc. Assembly was performed by Epigenetiks Ltd. Co. using Trinity (Grabherr et al., 2013) . Sequenceread files were retrieved in FASTQ format. Quality control was performed on FastQC (Andrews, no date). Blatella germanica and Zootermopsis nevadensis were chosen as the genomes closest to C. morosus. The assembly file was retrieved in FASTA format. Functional and structural annotations were performed via blastx against NCBI B. germanica sequences. The data were submitted to Sequence Read Archives (SRA7949781).

### 2.4. GPCRome prediction

In order to predict open reading frames (ORFs), the sequences were submitted to ORFPREDICTOR (Min et al., 2005) . All six ORFs were analyzed for the presence of TM helices in TMHMM (Krogh et al., 2001) . TMcontaining ORFs were separated into different files. The ORFs, which included more than 2 TM regions, were aligned with the NCBI BLASTp tool. Results of the top ten hits were taken and filtered according to the presence of GPCR domains. In order to check for the functional units and patterns of these sequences ExPASy (Gasteiger et al., 2003) , BLASTp, and SMART (Schultz et al., 1998) were used. Structural GPCR domains were determined in GPCRHMM (Wistrand et al., 2006) . Finally, putative GPCR classification was performed in 6 groups according to the nomenclature generated by G PCRdb (PándySzekeres et al., 2018 ).

### 2.5. cDNA synthesis

The amount of RNA of different tissues was adjusted to 1 µg. First strand cDNA synthesis was performed as recommended in the protocol of the SensiFAST cDNA Synthesis Kit (BIO-65054, BIOLINE, London, UK).

### 2.6. qPCR

The primers were designed from the putative GPCR transcripts via the BlastPrimer tool. Quantitative PCR reactions were prepared according to the recommendations of the SensiFAST SYBR No-ROX Kit (SF581-B054620, BIOLINE). Each sample was prepared in technical duplicates. The PCR efficiency of each primer set was calculated by the standard curve method taking the curves with R2 ≥ 0.99. Fold changes in expression levels were calculated via the REST method (including the PCR efficiencies) with regard to GAPDH as the reference gene and ovary tissue as the calibrator. The other tissues included in the analysis were the brain together with the endocrine glands corpora allata (CA) and corpora cardiaca (CC), ganglia, Malpighian tubules, crop and foregut, gastric cecum, postposterior midgut with the hindgut, fat body, and the aorta. Each qPCR reaction was performed in biological triplicates, each containing at least three animals. The specificity and consistency of the reactions were determined via RT-PCR.

## 3. Results

### 3.1. Novel GPCR sequences were predicted from the transcriptome of the adult stick insect

**Figure 1:**
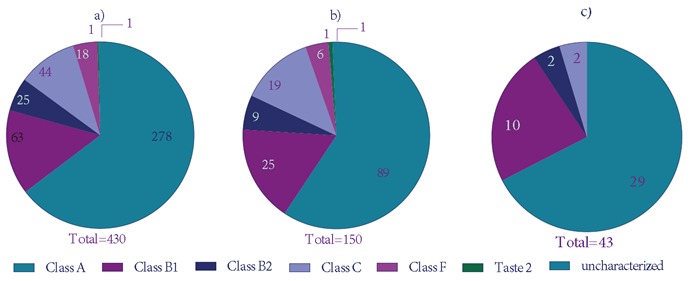
Distribution of putative GPCR transcripts in 6 different GPCR classes. a) All of the transcripts giving GPCR hits in blast search. b) The transcripts that yield highly significant (E ≤ 0.01) GPCR hits in blast search. c) The transcripts that yield highly significant GPCR hits in blast search and contain at least 7 helices in their ORFs.

The total body of adult female C. morosus specimens was used for total RNA sequencing and 94,820,114 base reads were obtained, which contributed to 128,397 transcripts within the assembled transcriptome. Bioinformatics analysis of this assembled transcriptome revealed 430 putative GPCR transcripts (Figure 1a). Blast results of these transcripts yielded a total of 150 transcripts giving highly significant similarity (E ≤ 0.01) with the known GPCRs and having 7TM conserved domains (Figure 1b). The maximum number of helices obtained in one transcript was 14 and these proteins mostly constitute the transporter proteins, which span the membrane several times (Dahl et al., 2004; Screpanti and Hunte, 2007) . GPCRs have 7 TM helices and some may have the 8th helix. Most of the sequences, which show only 1 TM helix, can come from the membrane anchorage region of the signal peptides (Hemminger et al., 1998) . Therefore, the ORFs with at least 7 helices were taken as the most probable GPCR transcripts (43 transcripts as shown in Figure 1c).

Classification of these putative GPCRs showed that one of the 150 highly significant GPCR transcripts was previously not characterized; however, we could only identify 3 TM helices of this novel GPCR. No full-length Frizzled or Taste-2 receptors could be obtained. Still, 1 Taste-2 and 3 Frizzled receptors were detected from partial transcripts. Types of GPCRs that are expressed in the adult C. morosus body can be seen in Table 1. No steroid or hydroxycarboxylic/nicotinic acid receptors were detected in the transcriptome, as expected for arthropods.

**Table 1:** The types of GPCRs that are obtained from the transcriptome of adult C. morosus body and their classification.

Type of GPCR	Subclass	Class
GPCR 143	Orphan	
5-Hydroxytryptamine Receptor	Aminergic Receptors	Class A
Adenosine Receptor	Nucleotide Receptors	Class A
Adipokinetic Hormone Receptor	Peptide Receptor	Class A
Allatostatin A Receptor	Peptide Receptor	Class A
Allatostatin C Receptor	Peptide Receptor	Class A
Alpha Adrenergic Receptor	Aminergic Receptors	Class A
Beta Adrenergic Receptor	Aminergic Receptors	Class A
Bombesin Receptor	Peptide Receptor	Class A
Cardioaccelatory Peptide Receptor	Vasopressin/Oxytocin Receptor	Class A
Cephalotocin Receptor	Vasopressin/Oxytocin Receptor	Class A
Chemokine Receptor	Protein Receptor	Class A
Cholecystokinin Receptor-Like	Peptide Receptor	Class A
Dopamine Receptor	Aminergic Receptors	Class A
Endothelin Receptor	Peptide Receptor	Class A
Fmrfamide Receptor	Peptide Receptor	Class A
Follicle-Stimulating Hormone Receptor	Peptide Receptor	Class A
Free Fatty Acid Receptor	Lipid Receptors	Class A
Glucose-Dependent Insulinotropic Receptor	Cannabinoid Receptor	Class A
Gonadotropin-Releasing Hormone II Receptor	Peptide Receptor	Class A
Histamine Receptor	Aminergic Receptors	Class A
Inotocin Receptor	Vasopressin/Oxytocin Receptor	Class A
Lutropin-Choriogonadotropic Hormone Receptor	Protein Receptor	Class A
Melanopsin	Sensory Receptors	Class A
Moody	(GPR84)	Class A
Muscarinic Acetylcholine Receptor	Aminergic Receptors	Class A
Neuromedin U Receptor	Peptide Receptor	Class A
Neuropeptide A10/Sex Peptide Receptor	Peptide Receptor	Class A
Neuropeptide A32 Receptor	Peptide Receptor	Class A
Neuropeptide A6a	Peptide Receptor	Class A
Neuropeptide Capa Receptor	Peptide Receptor	Class A
Neuropeptide Cchamide-1 Receptor	Peptide Receptor	Class A
Neuropeptide F Receptor	Peptide Receptor	Class A
Neuropeptide FF Receptor	Peptide Receptor	Class A
Neuropeptide Receptor	Peptide Receptor	Class A
Neuropeptide Receptor A27	Peptide Receptor	Class A
Neuropeptide Sifamide Receptor	Peptide Receptor	Class A
Neuropeptide Y Receptor	Peptide Receptor	Class A
Octopamine or Capa Receptor	Adrenoreceptor/Vasopressin	Class A
Octopamine Receptor	Adrenoreceptors	Class A
Odorant	Aminergic Receptors	Class A
Odorant Receptor	Odorant Receptor	Class A
Odorant Receptor 4	Odorant Receptor	Class A
Odorant Receptor 40	Sensory Receptor	Class A
Odorant Receptor 83a	Sensory Receptor	Class A
Opsin	Sensory Receptors	Class A
Prolactin-Releasing Peptide Receptor	Peptide Receptor	Class A
Relaxin Receptor	Peptide Receptor	Class A
Rfamide Receptor	Peptide Receptor	Class A
Rhodopsin	Sensory Receptors	Class A
Ryamide Receptor	Neuropeptide Y Receptor	Class A
Sex Peptide Receptor	Peptide Receptor	Class A
Sifamide Receptor	Peptide Receptor	Class A
Tachykinin-Like Peptides Receptor	Peptide Receptor	Class A
Thyrotropin Receptor	Protein Receptor	Class A
Trace Amine Associated Receptor	Aminergic Receptors	Class A
Tyramine Receptor	Adrenoreceptors	Class A
Vasopressin/Oxytocin Receptor	Peptide Receptor	Class A
Calcitonin Receptor	Peptide Receptor	Class B1
Diuretic Hormone Receptor	Peptide Receptor	Class B1
Mth-Like	Methuselah-Like	Class B1
PDF Receptor	VIP And PACAP Receptor	Class B1
Pigment Dispersing Factor Receptor	VIP And PACAP Receptor	Class B1
Adhesion GPCR G2	Adhesion Receptor	Class B2
Adhesion GPCR A3	Adhesion Receptor	Class B2
GABA-B Receptor	Amino Acid Receptor	Class C
Gustatory Receptor	Sensory Receptor	Class C
Gustatory Receptor 2	Sensory Receptor	Class C
Gustatory Receptor 28b	Sensory Receptor	Class C
Gustatory Receptor 43a	Sensory Receptor	Class C
Gustatory Receptor 64e	Sensory Receptor	Class C
Gustatory Receptor 64f	Sensory Receptor	Class C
Metabotropic Glutamate Receptor	Amino Acid Receptor	Class C
Frizzled	Frizzled Receptors	Class F
Frizzled-10	Frizzled Receptors	Class F
Gustatory Receptor 28a	Sensory Receptor	Taste 2

Within the highly significant and full-length ORFs of adult female C. morosus, there were at least 29 Class A, 10 Class B1, 2 Class B2, and 2 Class C GPCRs. Some of these GPCRs were chosen for tissue-specific expression analysis such as inotocin receptor (CamInoR), octopamine receptor (CamOctR), tyramine receptor 2-like (CamTyr2R), calcitonin gene-related peptide 1 receptor (CamCalR), and diuretic hormone receptor (CamDHR). Moreover, other receptors, which exhibit only partial transcripts, were included due to their relation to neuropeptide signaling, such as sex peptide receptor (CamSPR), allatostatin A and C receptors (CamAlstR-A and CamAlstR-C), neuropeptide Y receptor (CamNPYR), and cholecystokinin receptor-like (CamCCKR). Adhesion GPCR G2-like (CamAdgrG2), which belong to Class B2 receptors, was also included. Gustatory receptor for sugar taste 43a-like (CamGr43a) was included due to its partial transcripts from Class C GPCRs. However, no transcript of a Frizzled receptor (Class F) could be included due to its low expression levels (minimum Cp ≥ 30) with regard to GAPDH (data not shown). During the GPCR prediction, an uncharacterized receptor (Orphan GPCR) was detected with a partial transcript. In order to obtain preliminary information for future studies, this receptor transcript was also included in the analysis. The list of chosen receptors and their putative transcripts is given in Table 2.

**Table 2:** The types of GPCRs chosen for the tissue-specific expression analysis and their transcripts. In the presence of multiple isoforms, the primers were designed to amplify all of them.

Type of Receptor	Class of GPCR	Transcript Code	# of Isoforms
Octopamine Receptor	Class A	TRINITY_DN30951_c0_g1	1
Tyramine Receptor 2-like	Class A	TRINITY_DN31442_c1_g1	2
Allatostatin A Receptor	Class A	TRINITY_DN62595_c0_g1	1
Allatostatin C Receptor	Class A	TRINITY_DN42122_c0_g1	1
Inotocin Receptor	Class A	TRINITY_DN36849_c0_g1	7
Neuropeptide Y Receptor	Class A	TRINITY_DN21880_c0_g1	1
Sex Peptide Receptor	Class A	TRINITY_DN54154_c0_g1	1
Cholecystokinin Receptor-like	Class A	TRINITY_DN35009_c0_g2	3
Calcitonin Gene-Related Peptide Type 1 Receptor	Class B1	TRINITY_DN35728_c0_g1	1
Diuretic Hormone Receptor	Class B1	TRINITY_DN29760_c0_g1	1
Adhesion GPCR G2-like	Class B2	TRINITY_DN19522_c0_g1	1
Gustatory receptor for sugar taste 43a-like	Class C	TRINITY_DN34134_c0_g1	1
Orphan GPCR	Uncharacterized	TRINITY_DN65134_c0_g1	1

**Figure 2:**
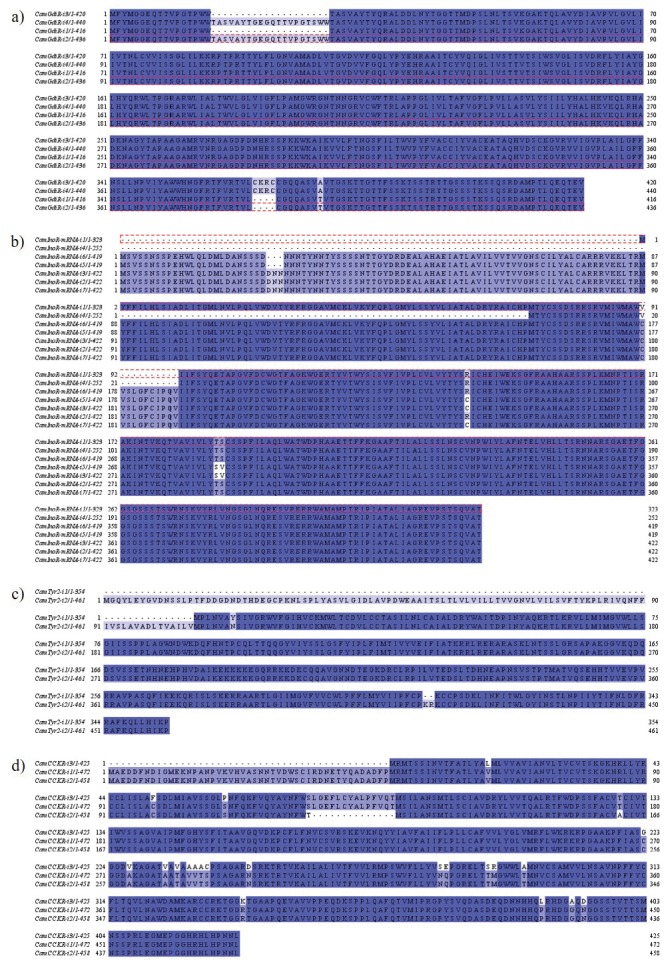
Representation of alignments of GPCR isoforms. Multiple alignment of each receptor sequence was retrieved from ClustalOmega and the colors were given according to % identity coloring of Jalview. Isoforms of a) CamGdiR, b) CamInoR, c) CamTyr2R, and d) CamCCKR were given.

Some GPCRs have more than one isoform with a few amino acid sequence variations. One of the most variable ones is the glucose-dependent insulinotropic receptor (CamGdiR) (Figure 2a). Four different receptor sequences have deletions in different parts of the receptors but these variations rely mainly on the N terminal or C terminal loops. CamInoRs and CamTyr2Rs show only one amino acid difference in their sequences (Figures 2b and 2c). On the other hand, CamCCKRs also showed deletions in some of the isoforms (Figure 2d).

### 3.2. Expression profiles of the predicted GPCRs in the adult stick insect body

**Figure 3:**
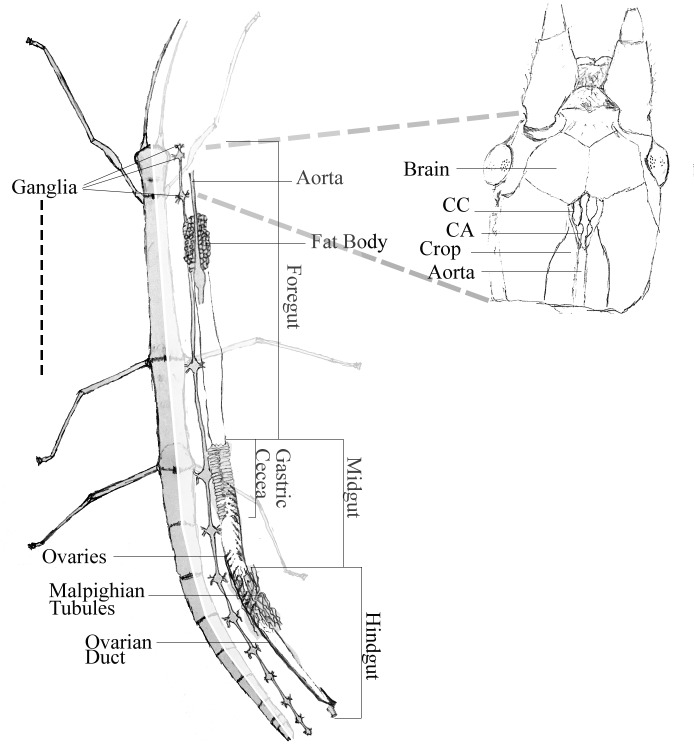
Representation of the anatomy of the adult C. morosus female. Only the organs that were included in RNA isolation are illustrated. CC: Corpus cadiacum, CA: Corpora allatum.

**Figure 4:**
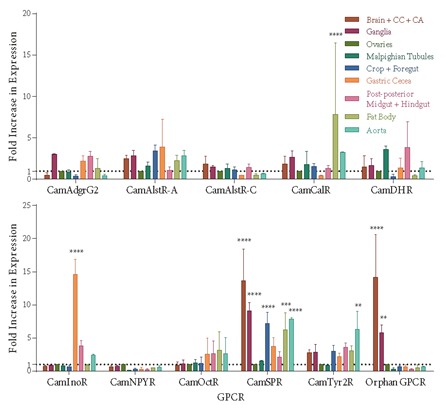
Fold change in expression of GPCR gene relative to GAPDH (= reference) in the ovary (= calibrator) via the REST method. The correlation within GPCR gene groups was calculated via two-way ANOVA test (*P ≤ 0.05, **P ≤ 0.01, ***P ≤ 0.001, and ****P ≤ 0.0001). N = 2 for Malpighian tubules and aorta samples, but 3 for the rest of the organs.

**Figure 5:**
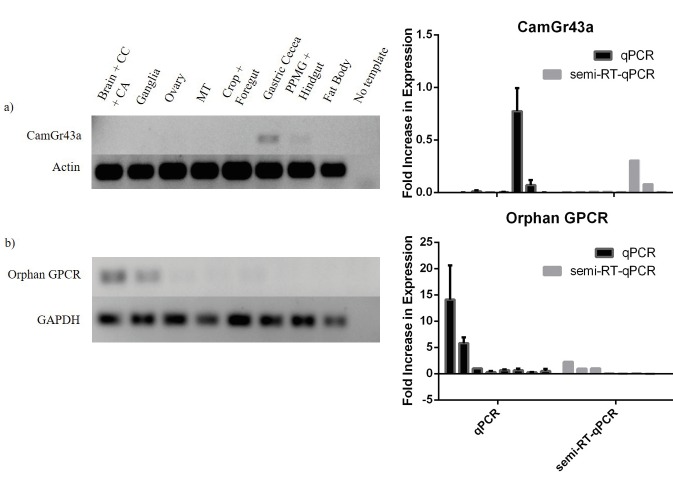
Comparison of semi-q RT-PCR and qPCR results of (a) CamGr43a and (b) Orphan GPCR. On the left part gel images of semi-q RT-PCR are given. The order of tissue samples was the same in both the gel images (left) and the graphs of both methods (right). The statistical analysis between the RT-PCR and qPCR graphs was performed in the Spearman correlation test. The graphs of Orphan GPCR were found to be correlated (P < 0.05).

In order to obtain tissue-specific expression profiles of these putative GPCR transcripts, 9 different parts of the adult animal were dissected (Figure 3). The brain was collected together with the neuroendocrine glands CA and CC. The gut of the animal was divided into three major parts: 1) crop together with the foregut, 2) gastric cecum with the anterior midgut inside, and 3) postposterior midgut together with the hindgut (Sh elomi et al., 2015 ). As seen in Figure 4, CamInoR, CamCalR, and CamTyr2R showed highly significant tissue-specific expression profiles. For instance, CamInoR was highly expressed in the gastric cecum, CamCalR in the fat body, and CamTyr2R in the aorta. Additionally, CamSPR was significantly expressed in 5 of the tissues compared to ovary levels, and its expression in the brain, CC, and CA was higher than in any of the other organs. On the other hand, expression profiles of some of the GPCRs (such as CamNPYR, CamAlstR-C, and CamOctR) showed a more uniform distribution in the body than other GPCRs. The efficiency of CamGr43a primers was insuficient in qPCR. Therefore, they were used in semiquantitative reverse transcription PCR (semi-q RTPCR) and the results were also compared with the qPCR data (Figure 5a). It was specifically expressed in gastric cecum and marginally expressed in postposterior midgut and hindgut samples. The most notable one was the brainspecific expression of the uncharacterized GPCR (Orphan GPCR), the function of which would be unraveled by further studies. Its expression was significantly higher in the brain, CC, and CA together with the ganglia than the other organs, more specifically higher than in the ganglia. This specific expression was also verified in semi-q RTPCR results (Figure 5b). The results of both methods were statistically correlated.

## 4. Discussion

GPCRs are responsible for various physiological functions in insects. Therefore, understanding the GPCR repertoire of an organism can facilitate understanding of a wide range of molecular mechanisms. Recently, the neuropeptidome repertoire of the stick insect C. morosus was published (Liessem et al., 2018) and most of those neuropeptides turned out to be ligands of GPCRs. However, the study did not focus on or reveal the GPCRs. Therefore, we aimed to incorporate the previously published neuropeptidome data into our GPCRome data in order to understand the physiological processes.

In our previous study, we identified a C-type of Allatostatin receptor (CamAlstR-C) from C. morosus (Duan Sahbaz et al., 2017) . AlstRs are the best-studied regulators of JH, which in turn is one of the most important hormones for insect development. However, not all of the AlstR types are expressed and function in the same way within all insect species. Therefore, understanding the expression profile of the different types of AlstR and ASTs would reveal the developmental and behavioral mechanisms specific of C. morosus . In our previous studies, we had difficulties in finding the ORF of other types of AlstRs via the same PCR-based techniques. Therefore, a genome- or transcriptome-wide search became a necessity to address this problem.

With the help of the tblastn tool, we could find out the partial mRNA sequences for AlstR-A and AlstR-C. The knowledge on the sequence of AlstR-B was very limited and it was used synonymously with the myoinhibitory peptide receptor (MIPR). uThs, we used the MIPR sequences to find a putative AlstR-B transcript in our transcriptome. Four partial transcripts matched our search query but each contained only one or two helices. A blastx search of these putative AlstR-B transcripts revealed that they were more similar with SPR than with the other MIPRs (data not shown). This was reasonable because the sex peptide and myoinhibitory peptide ligands differ from each other with the presence of one additional amino acid and they were shown to activate both receptors (Yamanaka et al., 2010) , meaning that these receptors can also be closely related. As a result of this, we decided to check the expression of the most reliable transcript, which has a significant similarity with other SPRs. Sex peptides are present in the ejaculate of the males and define the postmating rejection of remating in females. The expression of SPR in Helicoverpa moths was abundant in neural tissues and pheromone glands (Hanin et al., 2011) . The data reported by Liessem et al. support the presence of AST-A, allatotropin, myoinhibitory peptide, small neuropeptide F, and other peptides in the (i) frontal ganglion, which regulates the motility of the foregut, (ii) antennal lobe, which is part of the brain, and (iii) CC (Liessem et al., 2018b) . As they did not expect to detect sex peptide in the female samples, our data that reveal SPR in the samples of brain, CC, CA, and the ganglia are consistent with the sex of the samples and with their data on the localization of myoinhibitory peptide. In other studies, myoinhibitory peptides were shown to result in receptivity of mating through the same neuronal circuit that the sex peptide acts on (Jang et al., 2017) . Additionally, expression of SPR was significantly higher in the foregut than in the ovary, which could be activated by myoinhibitory peptides in the frontal ganglion.

Expression analyses of AlstR types did not yield tissue-specific profiles. The function of these receptors varies between different insect species. Neuropeptidome data showed that AST-A peptide was present in the head (antennal lobe) of C. morosus (Liessem et al., 2018) . AlstR-A of other insects is responsible for JH inhibition as well as regulation of feeding behavior, gut motility, and sleep behavior (Secher et al., 2001; Audsley and Weaver, 2009) . AlstR-C has roles similar to those of AlstR-A and AST-C was abundant in the frontal ganglion of C. morosus (Liessem et al., 2018) . In the mosquito, AlstR-C expression is high in the brain and abdominal ganglia. Although our results showed that this receptor was widely expressed, the highest expression was in the brain, CC, and CA organs, albeit insignificant. On the other hand, the highest expression of AlstR-A was in parts of the gut. Therefore, it is possible that the major regulator of JH might be AlstR-C but for gut motility, AlstR-A.

One of the unexpected results was the detection of an InoR in our analyses, despite the absence of inotocin peptide in C. morosus (Liessem et al., 2018) . CamInoR showed significantly high expression in the gastric cecum (with the anterior midgut inside). The inotocin peptide is the homolog of vasopressin/oxytocin family peptides and responsible for the social and reproductive behaviors of ants (Chérasse and Aron, 2017) . However, the gastric cecum is a part of the midgut and responsible for increasing the surface area of the midgut. Our result may indicate that this receptor has another task in this insect species, which would necessitate further studies.

Octopamine and tyramine receptors were expected to exhibit the highest expression in the CNS but they can also be detected in the intestine, muscles, Malpighian tubules, and other organs depending on their type ( ElKholy et al., 2015 ). The targets of octopamine neurons are the ovaries and oviducts. Therefore, their receptors are also present in reproductive organs and they control ovulation (Monastirioti, 2003) . CamOctR does not show tissue-specific expression but its expression is slightly higher in the gastric cecum, midgut, hindgut, aorta, and fat body than in other organs. On the other hand, CamTyr2R is highly expressed in the aorta. These results should be further analyzed in physiological studies.

DHR is the peptide receptor responsible for the water and ion homeostasis of insects (Paluzzi et al., 2010) . It was expected to be highly expressed in the Malpighian tubules, but the result was consistent but insignificant. Therefore, it may not be expressed in a tissue-specific manner but probably functions similarly in C. morosus as in other insect species. The second peptide receptor from Class B1 was CamCalR, which is expected to have roles in calcium metabolism. Some studies state that insect species express at least two CalR/DHR types and they differ in expression profiles and probably in their functions (Zandawala et al., 2013) . Our results show that expression of CamCalR is significantly higher in the fat body than in other organs. The difference between these two receptors should be further analyzed.

There are not many studies on the expression of adhesion GPCRs in insects. However, human adhesion GPCR G2 is mostly expressed in male reproductive organs together with adipose tissue (Regard et al., 2009) . Our results reveal that CamAdgrG2 is not expressed in a tissue-specific manner but has slightly higher levels of expression in the ganglia, gastric cecum, midgut, and hindgut.

CCKR or tachykinin receptors are known to be expressed in the CNS and gut (Xu et al., 2016) . Our results support its expression in the gut but do not show a gutspecific expression profile. NPYR receptors have roles in regulating appetite and circadian rhythm (Kokot and Ficek, 1999; Liesch et al., 2013) . It seems that CamNPYR is similarly expressed in different tissues. Although the difference is not significant, its highest expression was in the ovaries, then in the ganglia and brain together with the neuroendocrine glands. Neuropeptidome analysis by Liessem et al. showed that the ligand of NPYR called small neuropeptide F (sNPF) was abundant in the CC gland that could be activating the receptor in the target organs, which we obtained in our expression analysis.

The most interesting result of this study is the prediction and detection of a CNS-specific orphan GPCR, which can be studied further.

Finally, this study revealed at least 43 GPCRs expressed in adult C. morosus and tissue expression profiles of some of them, which in turn can facilitate further GPCR studies on C. morosus.

## Acknowledgments

This study was funded by Boğaziçi University Scientific Research Projects (BAP 17B01D1). The authors thank Prof Aslı Tolun, Boğaziçi University, for providing the LightCycler, Prof O Uğur Sezerman, Acıbadem University, for his assistance during the in silico analyses, Assoc Prof İbrahim Yaman for his assistance with the expression analyses, and Gizem Doğan Marangoz for her help with the anatomical illustrations. ·
